# Some inequalities involving the extended gamma function and the Kummer confluent hypergeometric *k*-function

**DOI:** 10.1186/s13660-018-1717-8

**Published:** 2018-06-19

**Authors:** Kottakkaran Sooppy Nisar, Feng Qi, Gauhar Rahman, Shahid Mubeen, Muhammad Arshad

**Affiliations:** 1 Department of Mathematics, College of Arts and Science at Wadi Aldawaser, Prince Sattam Bin Abdulaziz University, Riyadh Region, Kingdom of Saudi Arabia; 2 Institute of Mathematics, Henan Polytechnic University, Jiaozuo, China; 3 Department of Mathematics, College of Science, Tianjin Polytechnic University, Tianjin, China; 4 Institute of Fundamental and Frontier Sciences, University of Electronic Science and Technology of China, Chengdu, China; 5 Department of Mathematics, International Islamic University, Islamabad, Pakistan; 6 Department of Mathematics, University of Sargodha, Sargodha, Pakistan

**Keywords:** 33B15, 33C15, 33B20, 33C05, 41A60, Extended gamma function, Inequality, Logarithmic convexity, Confluent hypergeometric *k*-function

## Abstract

In the paper, the authors present some inequalities involving the extended gamma function and the Kummer confluent hypergeometric *k*-function via some classical inequalities such as Chebychev’s inequality for synchronous (or asynchronous, respectively) mappings, give a new proof of the log-convexity of the extended gamma function by using the Hölder inequality, and introduce a Turán type mean inequality for the Kummer confluent *k*-hypergeometric function.

## Introduction

The gamma function Γ can be defined [24, 28, 31, 32] by

$$ \Gamma(z)= \int_{0}^{\infty}t^{z-1}e^{-t} \,\mathrm{d}t,\quad\Re(z)>0. $$

Alternatively, it can also be defined [20] by

$$ \Gamma(z)=\lim_{n\to\infty}\frac{n!n^{z-1}}{(z)_{n}}, $$

where $(z)_{n}$ for $z\ne0$ is the Pochhammer symbol defined [27] as

$$ (z)_{n}= \textstyle\begin{cases} z(z+1)(z+2)\cdots(z+n-1), & n\ge1;\\ 1, & n=0. \end{cases} $$

The relation between $(z)_{n}$ and $\Gamma(z)$ is

$$ (z)_{n}=\frac{\Gamma(z+n)}{\Gamma(z)}. $$

The beta function $B(x,y)$ can be defined [18, 21, 22] by

$$ B(x,y)= \int_{0}^{1}t^{x-1}(1-t)^{y-1} \,\mathrm{d}t,\quad\Re(x),\Re(y)>0 $$

and can be expressed by

$$ B(x,y)=\frac{\Gamma(x)\Gamma(y)}{\Gamma(x+y)},\quad\Re(x),\Re(y)>0. $$

In 1995, Chaudhry and Zubair [4] introduced the extended gamma function,

1.1 $$ \Gamma_{b}(z)= \int_{0}^{\infty}t^{z-1}e^{-t-bt^{-1}} \,\mathrm{d}t,\quad\Re(z)>0, b\ge0. $$

If $b=0$, then $\Gamma_{b}$ becomes the classical gamma function Γ.

In 1997, Chaudhry et al. [3] introduced the extended beta function,

$$ B_{b}(x,y)= \int_{0}^{1}t^{x-1}(1-t)^{y-1}e^{-b/t(1-t)} \,\mathrm{d}t,\quad\Re(b),\Re(x),\Re(y)>0. $$

It is clear that $B_{0}(x,y)=B(x,y)$.

In 2009, Barnard et al. [1] established three inequalities

$$\begin{aligned}& \bigl[\phi(a, a+b,x)\bigr]^{2}>\phi(a+v,a+b,x)\phi(a-v,a+b,x), \\& \bigl[\phi(a, c,x)\bigr]^{2}>\phi(a+v,c,x)\phi(a-v,c,x), \end{aligned}$$

and

$$\begin{aligned} A\bigl(\phi(a+v, a+b,x),\phi(a-v,a+b,x)\bigr)&>\phi(a,a+b,x) \\ &> G\bigl(\phi(a+v,a+b,x),\phi(a-v,a+b,x)\bigr), \end{aligned}$$

where $A(\alpha,\beta)=\frac{\alpha+\beta}{2}$ and $G(\alpha,\beta )=\sqrt{\alpha\beta}$ are the arithmetic and geometric means and

$$ \phi(a,b,x)=\sum_{n=0}^{\infty} \frac{(a)_{n}}{(b)_{n}}\frac{x^{n}}{n!} $$

is the Kummer confluent hypergeometric function [25, 28].

The Kummer confluent hypergeometric *k*-function is defined by

$$ \phi_{k}(a,b,x)=\sum_{n=0}^{\infty} \frac{(a)_{n,k}}{(b)_{n,k}}\frac{x^{n}}{n!}, $$

where

$$ (a)_{n,k}=a(a+k) (a+2k)\cdots\bigl[a+(n-1)k\bigr] $$

for $n\ge1$ and $k>0$ with $(a)_{0,k}=1$ is the Pochhammer *k*-symbol, which can also be rewritten as

$$ (a)_{n,k}=\frac{\Gamma_{k}(a+nk)}{\Gamma_{k}(a)} $$

and the gamma *k*-function $\Gamma_{k}(a)$ is defined [6] by

$$ \Gamma_{k}(a)= \int_{0}^{\infty}t^{a-1}e^{-t^{k}/k} \,\mathrm{d}t. $$

In 2012, Mubeen [15] introduced the *k*-analogue of Kummer’s transformation as

1.2 $$ \phi_{k}(a,b,x)=e^{x}\phi _{k}(a,b-a,-x). $$

In Sect. 2, we prepare two lemmas. In Sect. 3, we discuss applications of some integral inequalities such as Chebychev’s integral inequality. In Sect. 4, we prove the logarithmic convexity of the extended gamma function. In the last section, we introduce a mean inequality of Turán type for the Kummer confluent hypergeometric *k*-function.

## Lemmas

In order to obtain our main results, we need the following lemmas.

### Lemma 2.1

(Chebychev’s integral inequality [7, 8, 12, 23])

*Let* $f,g,h:I\subseteq\mathbb{R}\to\mathbb{R}$ *be mappings such that* $h(x)\ge0$, $h(x)f(x)g(x)$, $h(x)f(x)$, *and* $h(x)g(x)$ *are integrable on I*. *If* $f(x)$ *and* $g(x)$ *are synchronous* (*or asynchronous*, *respectively*) *on* *I*, *that is*,

$$ \bigl[f(x)-f(y)\bigr] \bigl[g(x)-g(y)\bigr]\gtreqless0 $$

*for all* $x,y\in I$, *then*

$$ \int_{I}h(x)\,\mathrm{d}x \int_{I}h(x)f(x)g(x)\,\mathrm{d}x\gtreqless \int_{I}h(x)f(x)\,\mathrm{d}x \int_{I}h(x)g(x)\,\mathrm{d}x. $$

### Lemma 2.2

(Hölder’s inequality [29, 30])

*Let* *p* *and* *q* *be positive real numbers such that* $\frac{1}{p}+\frac {1}{q}=1$ *and* $f,g:[c,d]\to\mathbb{R}$ *be integrable functions*. *Then*

$$ \biggl\vert \int_{c}^{d}f(x)g(x)\,\mathrm{d}x \biggr\vert \le \biggl[ \int_{c}^{d} \bigl\vert f(x) \bigr\vert ^{p}\,\mathrm{d}x \biggr]^{1/p} \biggl[ \int_{c}^{d} \bigl\vert g(z) \bigr\vert ^{q}\,\mathrm{d}x \biggr]^{1/q}. $$

## Inequalities involving the extended gamma function via Chebychev’s integral inequality

In this section, we prove some inequalities involving the extended gamma function via Chebychev’s integral inequality in Lemma 2.1.

### Theorem 3.1

*Let* *m*, *p* *and* *r* *be positive real numbers such that* $p>r>-m$. *If* $r(p-m-r)\gtreqless0$, *then*

3.1 $$ \Gamma_{b}(m)\Gamma_{b}(p)\gtreqless\Gamma _{b}(p-r)\Gamma_{b}(m+r). $$

### Proof

Let us define the mappings $f,g,h:[0,\infty)\rightarrow[0,\infty)$ given by

$$ f(t)=t^{p-r-m},\quad\quad g(t)=t^{r},\quad\text{and}\quad h(t)=t^{m-1}e^{-t-bt^{-1}}. $$

If $r(p-m-r)\gtreqless0$, then we can claim that the mappings *f* and *g* are synchronous (asynchronous) on $(0,\infty)$. Thus, by applying Chebychev’s inequality on $I=(0,\infty)$ to the functions *f*, *g* and *h* defined above, we can write

$$\begin{aligned}& \int_{0}^{\infty}t^{m-1}e^{-t-bt^{-1}} \,\mathrm{d}t \int_{0}^{\infty}t^{p-r-m}t^{r}t^{m-1}e^{-t-bt^{-1}} \,\mathrm{d}t \\& \quad \gtreqless \int_{0}^{\infty}t^{p-r-m}t^{m-1}e^{-t-bt^{-1}} \,\mathrm{d}t \int_{0}^{\infty}t^{r}t^{m-1}e^{-t-bt^{-1}} \,\mathrm{d}t. \end{aligned}$$

This implies that

$$\begin{aligned}& \int_{0}^{\infty}t^{m-1}e^{-t-bt^{-1}} \,\mathrm{d}t \int_{0}^{\infty}t^{p-1}e^{-t-bt^{-1}} \,\mathrm{d}t \\& \quad \gtreqless \int_{0}^{\infty}t^{p-r-1}e^{-t-bt^{-1}} \,\mathrm{d}t \int_{0}^{\infty}t^{m+r-1}e^{-t-bt^{-1}} \,\mathrm{d}t. \end{aligned}$$

By (1.1), we acquire the required inequality (3.1). □

### Corollary 3.1

*If* $p>0$ *and* $q\in\mathbb{R}$ *with* $\vert q \vert < p$, *then*

$$ \Gamma_{b}(p)\leq\bigl[\Gamma_{b}(p-q)\Gamma _{b}(p+q)\bigr]^{1/2}. $$

### Proof

By setting $m=p$ and $r=q$ in Theorem 3.1, we obtain $r(p-m-r)=-q^{2}\leq0$ and then the inequality (3.1) provides the desired Corollary 3.1. □

### Theorem 3.2

*If* $m, n>0$ *are similarly* (*oppositely*) *unitary*, *then*

$$ \Gamma_{b}(m+n+b)\gtreqless\frac{\Gamma_{b}(m+b+1)\Gamma _{b}(n+b+1)}{\Gamma_{b}(b+2)}. $$

### Proof

Consider the mappings $f,g,h:[0,\infty)\rightarrow[0,\infty)$ defined by

$$ f(t)=t^{m-1},\quad\quad g(t)=t^{n-1}, \quad\text{and}\quad h(t)=t^{b+1}e^{-t-bt^{-1}}. $$

Now if the condition $(m-1)(n-1)\gtreqless0$ holds, then Chebychev’s integral inequality applied to the functions *f*, *g*, and *h* means

$$\begin{aligned}& \int_{0}^{\infty}t^{b+1}e^{-t-bt^{-1}} \,\mathrm{d}t \int_{0}^{\infty}t^{m-1}t^{n-1}t^{b+1}e^{-t-bt^{-1}} \,\mathrm{d}t \\& \quad \gtreqless \int_{0}^{\infty}t^{m-1}t^{b+1}e^{-t-bt^{-1}} \,\mathrm{d}t \int_{0}^{\infty}t^{n-1}t^{b+1}e^{-t-bt^{-1}} \,\mathrm{d}t. \end{aligned}$$

This implies that

$$\begin{aligned}& \int_{0}^{\infty}t^{b+1}e^{-t-bt^{-1}} \,\mathrm{d}t \int_{0}^{\infty}t^{m+n+b-1}e^{-t-bt^{-1}} \,\mathrm{d}t \\& \quad \gtreqless \int_{0}^{\infty}t^{m+b}e^{-t-bt^{-1}} \,\mathrm{d}t \int_{0}^{\infty}t^{n+b}e^{-t-bt^{-1}} \,\mathrm{d}t. \end{aligned}$$

By the definition of the extended gamma function, we have

$$ \Gamma_{b}(b+2)\Gamma_{b}(m+n+b)\gtreqless\Gamma _{b}(m+b+1)\Gamma_{b}(n+b+1), $$

or

$$ \Gamma_{b}(m+n+b)\gtreqless\frac{\Gamma_{b}(m+b+1)\Gamma _{b}(n+b+1)}{\Gamma_{b}(b+2)}. $$

The required proof is complete. □

### Corollary 3.2

*If* $b=0$, *then*

$$ \Gamma(m+n)\gtreqless{mn\Gamma(m)\Gamma(n)}. $$

### Theorem 3.3

*If* *m* *and* *n* *are positive real numbers such that* *m* *and* *n* *are similarly* (*oppositely*) *unitary*, *then*

$$ \Gamma_{b}(b+1)\Gamma_{b}(m+n+b+1)\gtreqless\Gamma _{b}((m+b+1)\Gamma_{b}(n+b+1), \quad b\geq0. $$

### Proof

Consider the mappings $f,g,h:[0,\infty)\rightarrow[0,\infty)$ defined by

$$ f(t)=t^{m},\quad\quad g(t)=t^{n}, \quad\text{and}\quad h(t)=t^{b}e^{-t-bt^{-1}}. $$

If the conditions of Theorem 3.1 hold, then the mappings *f* and *g* are synchronous (asynchronous) on $[0,\infty)$. Thus, by applying Chebychev’s integral inequality in Lemma 2.1 to the functions *f*, *g* and *h* defined above, we have

$$\begin{aligned}& \int_{0}^{\infty}t^{b}e^{-t-bt^{-1}} \,\mathrm{d}t \int_{0}^{\infty}t^{m}t^{n}t^{b}e^{-t-bt^{-1}} \,\mathrm{d}t \\& \quad \gtreqless \int_{0}^{\infty}t^{m}t^{b}e^{-t-bt^{-1}} \,\mathrm{d}t \int_{0}^{\infty}t^{n}t^{b}e^{-t-bt^{-1}} \,\mathrm{d}t. \end{aligned}$$

This implies that

$$\begin{aligned}& \int_{0}^{\infty}t^{b}e^{-t-bt^{-1}} \,\mathrm{d}t \int_{0}^{\infty}t^{m+n+b}e^{-t-bt^{-1}} \,\mathrm{d}t \\& \quad \gtreqless \int_{0}^{\infty}t^{m+b}e^{-t-bt^{-1}} \,\mathrm{d}t \int_{0}^{\infty}t^{n+b}e^{-t-bt^{-1}} \,\mathrm{d}t. \end{aligned}$$

Thus by the definition of extended gamma function, we have

$$ \Gamma_{b}(b+1)\Gamma_{b}(m+n+b+1)\gtreqless\Gamma _{b}(m+b+1)\Gamma_{b}(n+b+1). $$

The required proof is complete. □

### Corollary 3.3

*If* $b=0$, *then*

$$ \Gamma(m+n)\gtreqless\frac{mn\Gamma(m)\Gamma(n)}{m+n}. $$

## Log-convexity of the extended gamma function

It is well known that, if $f>0$ and ln*f* is convex, then *f* is said to be a logarithmically convex function. Every logarithmically convex must be convex. See [16] and [19, Remark 1.9]. In this section, we verify the log-convexity of extended gamma function.

### Theorem 4.1

*The extended gamma function* $\Gamma_{b}:(0,\infty)\to \mathbb{R}$ *is logarithmically convex*.

### Proof

Let *p* and *q* be positive numbers such that $\frac{1}{p}+\frac {1}{q}=1$. Since

$$ \Gamma_{b} \biggl(\frac{x}{p}+\frac{y}{q} \biggr)\le \bigl[\Gamma_{b}(x)\bigr]^{1/p}\bigl[\Gamma _{b}(y)\bigr]^{1/q}, $$

see [5], letting $\lambda=\frac{1}{p}$ and $(1-\lambda)=\frac {1}{q}$ leads to

$$ \Gamma_{b}\bigl[\lambda x+(1-\lambda)y\bigr]\le\bigl[\Gamma _{b}(x)\bigr]^{\lambda}\bigl[\Gamma_{b}(y) \bigr]^{(1-\lambda)}. $$

As a result, the function $\Gamma_{b}$ is logarithmically convex. □

## A mean inequality of the Turán type for the Kummer confluent hypergeometric *k*-function

In this section, we present a mean inequality involving the confluent hypergeometric *k*-function. For this purpose, we consider the relation

5.1 $$ \phi_{k}(a+k,b,x)-\phi_{k}(a,b,x)= \frac{kx}{b}\phi_{k}(a+k,b+k,x), \quad k>0. $$

### Theorem 5.1

*For* $a,b, k>0$ *and* $v\in\mathbb{N}$ *with* $a,b\ge v-k$, *the inequality*

5.2 $$ \bigl[\phi_{k}(a,a+b,x)\bigr]^{2}>\phi _{k}(a+v,a+b,x)\phi_{k}(a-v,a+b,x) $$

*is valid for all nonzero* $x\in\mathbb{R}$.

### First proof

Assume that $x>0$. For $c\ne0,-1,-2,\ldots$ , define

$$ f_{v,k}(x)=\bigl[\phi_{k}(a,c,x)\bigr]^{2}-\phi _{k}(a+v,c,x)\phi_{k}(a-v,a+b,x) $$

and

$$ f_{v+k,k}(x)=\phi_{k}(a,c,x)^{2}-\phi _{k}(a+v+k,c,x)\phi_{k}(a-v-k,a+b,x). $$

From (5.1), it follows that

$$\begin{aligned} f_{v+k,k}(x)-f_{v,k}(x)&=\phi_{k}(a+v,c,x)\phi _{k}(a-v,c,x) \\ &\quad{} -\phi_{k}(a+v+k,c,x)\phi_{k}(a-v-k,c,x) \\ &=\phi_{k}(a-v,c,x)\bigl[\phi_{k}(a+v,c,x)-\phi _{k}(a+v+k,c,x)\bigr] \\ &\quad{} +\phi_{k}(a+v+k,c,x)\bigl[\phi_{k}(a-v,c,x)-\phi _{k}(a-v-k,c,x)\bigr] \\ &=\phi_{k}(a-v,c,x) \biggl(\frac{-kx}{c} \biggr)\phi _{k}(a+v+k,c+k,x) \\ &\quad{} +\phi_{k}(a+v+k,c,x) \biggl(\frac{kx}{c} \biggr)\phi _{k}(a-v,c+k,x) \\ &=\frac{kx}{c}g_{v,k}(x), \end{aligned}$$

where

$$\begin{aligned} g_{v,k}(x)&=\phi_{k}(a+v+k,c,x)\phi_{k}(a-v,c+k,x) \\ &\quad{} -\phi_{k}(a-v,c,x)\phi_{k}(a+v+k,c+k,x). \end{aligned}$$

Accordingly, by the Cauchy product, we have

$$\begin{aligned} g_{v,k}(x)&=\sum_{s=0}^{\infty}\sum _{r=0}^{s}\frac{(a+v+k)_{s,k}(a-v)_{s-r,k}}{r!(s-r)!} \\ &\quad{} \times\biggl[\frac{1}{(c)_{s,k}(c+k)_{s-r,k}}-\frac {1}{(c)_{s-r,k}(c+k)_{s,k}} \biggr]x^{s} \\ &=\sum_{s=0}^{\infty}\sum _{r=0}^{s}\frac{(a+v+k)_{s,k}(a-v)_{s-r,k}}{r!(s-r)!} \biggl[ \frac{(c+mk)-(c+nk-mk)}{c(c+k)_{s,k}(c+k)_{s-r,k}} \biggr]x^{s} \\ &=\frac{k}{c}\sum_{s=0}^{\infty}\sum _{r=0}^{s}T_{s,r,k}(2r-s)x^{s}, \end{aligned}$$

where

$$ T_{s,r,k}=\frac{(a+v+k)_{s,k}(a-v)_{s-r,k}}{r!(s-r)!(c+k)_{s,k}(c+k)_{s-r,k}}. $$

If *s* is even, then

$$\begin{aligned} \sum_{r=0}^{s}T_{s,r,k}(2r-s)&=\sum _{r=0}^{s/2-1}T_{s,r,k}(2r-s) +\sum _{r=s/2+1}^{s}T_{s,r,k}(2r-s) \\ &=\sum_{r=0}^{s/2-1}T_{s,r,k}(2r-s)+ \sum_{r=0}^{s/2-1}T_{s,s-r,k}\bigl(2(s-r)-s \bigr) \\ &=\sum_{r=0}^{\lceil(s-1)/2\rceil}(T_{s,s-r,k}-T_{s,r,k}) (s-2r), \end{aligned}$$

where $\lceil x\rceil$ denotes the ceiling function whose value is the greatest integer not more than *x*. Similarly, if *s* is odd,

$$ \sum_{r=0}^{s}T_{s,r,k}(2r-s)=\sum _{r=0}^{\lceil(s-1)/2\rceil}(T_{s,s-r,k}-T_{s,r,k}) (s-2r). $$

Accordingly,

5.3 $$ \begin{aligned}[b] f_{v+k,k}(x)-f_{v,k}(x)&=\frac{kx}{c}g_{v,k}(x) \\ &=\frac{k^{2}x}{c^{2}} \sum_{s=1}^{\infty} \sum _{r=0}^{\lceil(s-1)/2\rceil}(T_{s,s-r,k}-T_{s,r,k}) (s-2r)x^{s}. \end{aligned} $$

Carefully simplifying gives

5.4 $$ \begin{aligned}[b]T_{s,s-r,k}-T_{s,r,k}&=\frac {(a+v+k)_{s,k}(a-v)_{s,k}-(a+v+1)_{s,k} (a-v)_{s,k}}{r!(s-r)!(c+k)_{s-r,k}(c+k)_{s,k}} \\ &=\frac{(a+v+k)_{s,k}(a-v)_{s,k}}{r!(s-r)!(c+k)_{s-r,k}(c+k)_{s,k}} \biggl[\frac{(a+v+k)_{s-r,k}}{(a+v+k)_{s,k}}-\frac {(a-v)_{s-r,k}}{(a-v)_{s,k}} \biggr] \\ &=\frac{(a+v+k)_{s,k}(a-v)_{s,k}}{r!(s-r)!(c+k)_{s-r,k}(c+k)_{s,k}}\bigl[h_{k}(a+v+k)-h_{k}(a-v)\bigr], \end{aligned} $$

where $h_{k}(x)=\frac{(x)_{s-r,k}}{(x)_{s,k}}$. For $x>0$ and $s-r>r$, that is, $[\frac{s-1}{2}]\ge r$, the logarithmic derivatives of $h_{k}$ is

$$ \frac{h_{k}'(x)}{h_{k}(x)}=\psi_{k}\bigl(x+(s-r)k\bigr)-\psi _{k}(x+nk)>0, $$

where $\psi_{k}=\frac{\Gamma_{k}'}{\Gamma_{k}}$ is the digamma *k*-function (see [6, 11, 16]). Hence, the function $h_{k}$ is increasing under the condition stated. This fact together with the aid of (5.3) and (5.4) yields

5.5 $$ \begin{aligned}[b] f_{v+k,k}(x)-f_{v,k}(x)&=\frac{kx}{c}g_{v,k}(x) \\ &=\frac{k^{2}x}{c^{2}} \sum_{s=1}^{\infty} \sum _{r=0}^{\lceil(s-1)/2\rceil} (T_{s,s-r,k}-T_{s,r,k}) (s-2r)x^{s}>0, \end{aligned} $$

where $a\ge v\ge0$, $x>0$, $c+k>0$, and $c\ne0$. Consequently, from (5.5), it follows that

$$ f_{v+k,k}(x)=\bigl[f_{v+k,k}(x)-f_{v,k}(x)\bigr]+ \bigl[f_{v,k}(x)-f_{v-k,k}(x)\bigr]+\cdots+\bigl[f_{1,k}(x)-f_{0,k}(x) \bigr] $$

is positive for $a\ge v\ge v-k\ge v-2k\ge\cdots\ge0$ and $f_{0,k}(x)=0$. Now replacing *v* by $v-k$ shows that

5.6 $$ f_{v,k}(x)>0,\quad x>0, v\in\mathbb{N}, a\ge v-k. $$

Therefore, the function $f_{v,k}$ is absolutely monotonic on $(0,\infty )$, that is, $f_{v,k}^{(\ell)}(x)>0$ for $\ell=0,1,2,\ldots$ . This proves Theorem 5.1 for the case $x>0$.

Now suppose that $x<0$, $a,b>0$, and $v\in\mathbb{N}$ with $a,b\ge v-k$. Since $\phi_{k}(a,c,x)$ is symmetric in *a* and *b*, by interchanging *a* and *b* in Theorem 5.1, we obtain

$$ \phi_{k}(b,a+b,-x)^{2}-\phi_{k}(b+v,a+b,-x) \phi_{k}(b-v,a+b,-x)>0. $$

By using Kummer’s transformation (1.2), we have

$$ e^{-2x}\phi_{k}(a,a+b,x)^{2}-e^{-2x} \phi_{k}(a-v,a+b,x)\phi_{k}(a+v,a+b,x)>0. $$

Thus, Theorem 5.1 also holds for $x<0$. □

### Second proof

Since

$$\begin{aligned} (a)_{n,k}&=a(a+k) (a+2k)\cdots\bigl(a+(n-1)k\bigr) \\ &=k^{n}\frac{a}{k} \biggl(\frac{a}{k}+1 \biggr) \biggl( \frac{a}{k}+2 \biggr)\cdots\biggl(\frac{a}{k}+(n-1) \biggr) \\ &=k^{n} \biggl(\frac{a}{k} \biggr)_{n}, \end{aligned}$$

it follows that

$$ \phi_{k}(a,b;x)=\sum_{n=0}^{\infty} \frac{(a)_{n,k}}{(b)_{n,k}}\frac{x^{n}}{n!} =\sum_{n=0}^{\infty} \frac{k^{n}(a/k)_{n}}{k^{n}(b/k)_{n}}\frac{x^{n}}{n!} =\phi\biggl(\frac{a}{k}, \frac{b}{k},x \biggr). $$

Replacing *a* and *b* by $\frac{a}{k}$ and $\frac{b}{k}$, respectively, gives Theorem 5.1. □

### Corollary 5.1

*If* $a>0$ *and* $c+k>0$ *with* $c\ne0$, *then the inequality*

$$ \bigl[\phi_{k}(a,c,x)\bigr]^{2}\ge\phi_{k}(a-v,c,x)\phi _{k}(a+v,c,x) $$

*holds for any* $v\in\mathbb{N}$ *with* $a\ge v-k$.

### Proof

This follows directly from the proof of Theorem 5.1 and the fact that Eq. (5.6) holds under the conditions $c+k>0$ and $c\ne0$. □

### Corollary 5.2

*If* $v\in\mathbb{N}$ *and* $a,b\ge v$, *then*

5.7 $$ \begin{aligned}[b] A\bigl(\phi_{k}(a+v,a+b,x),\phi_{k}(a-v,a+b,x) \bigr)&>\phi_{k}(a,a+b,x) \\ &>G\bigl(\phi_{k}(a+v,a+b,x),\phi_{k}(a-v,a+b,x)\bigr) \end{aligned} $$

*for all nonzero* $x\in\mathbb{R}$, *where* *A* *and* *G* *are*, *respectively*, *the arithmetic and geometric means*.

### Proof

First assume $x\ge0$ and $a,b\ge v$ for $v\in\mathbb{N}$. Then the left hand side inequality in (5.7) is a direct consequence of the facts that

$$ A\bigl((a+v)_{s,k},(a-v)_{s,k}\bigr)=(a)_{s,k} $$

for $s=0,1$ and

$$ A\bigl((a+v)_{s,k},(a-v)_{s,k}\bigr)>(a)_{s,k} $$

for $s\ge2$. Hence, by induction, we have

$$\begin{aligned} A\bigl(\phi_{k}(a+v,a+b,x),\phi_{k}(a-v,a+b,x)\bigr) &= \sum_{s=0}^{\infty}\frac{A((a+v)_{s,k},(a-v)_{s,k})x^{s}}{(a+b)_{s,k}s!} \\ &>\sum_{s=0}^{\infty}\frac{(a)_{s,k}x^{s}}{(a+b)_{s,k}s!}=\phi _{k}(a, a+b,x). \end{aligned}$$

For $x\ge0$, the right hand side inequality in (5.7) follows from taking square root of (5.2). The proof of Corollary 5.2 for $x\ge0$ is thus complete.

Now assume $x<0$ with $a,b\ge v$. Interchanging *a* and *b* in (5.7) one arrives at

$$\begin{aligned} A\bigl(\phi_{k}(b+v,a+b,-x),\phi_{k}(b-v,a+b,-x)\bigr) &> \phi_{k}(b,a+b,-x) \\ &>G\bigl(\phi_{k}(b+v,a+b,-x),\phi_{k}(b-v,a+b,-x)\bigr). \end{aligned}$$

Making use of the *k*-analogue of Kummer’s transformation and the homogeneity of *A* and *G* acquires

$$\begin{aligned} e^{-x}A\bigl(\phi_{k}(a-v,a+b,x),\phi_{k}(a+v,a+b,x) \bigr)&>e^{-x}\phi_{k}(a,a+b,x) \\ & >e^{-x}G\bigl(\phi_{k}(a-v,a+b,x),\phi _{k}(a+v,a+b,x)\bigr). \end{aligned}$$

Consequently, Theorem (5.7) also follows for $x<0$. □

### Remark 5.1

In Sect. 5, we have established a Turán type and mean inequality for *k*-analogue of the Kummer confluent hypergeometric function. If we let $k\to1$, then we can conclude to the corresponding inequalities of the confluent hypergeometric function.

### Remark 5.2

In [2], some inequalities of the Turán type for confluent hypergeometric functions of the second kind were also discovered.

### Remark 5.3

By the way, we note that Refs. [9, 10, 13, 14, 26, 32, 33] belong to the same series in which inequalities and complete monotonicity for functions involving the gamma function $\Gamma(x)$ and the logarithmic function $\ln(1+x)$ were discussed.

### Remark 5.4

This paper is a slightly revised version of the preprint [17].

## Conclusions

In this paper, we present some inequalities involving the extended gamma function $\Gamma_{b}(z)$ via some classical inequalities such as Chebychev’s inequality for synchronous (or asynchronous, respectively) mappings, give a new proof of the log-convexity of the extended gamma function $\Gamma_{b}(z)$ by using the Hölder inequality, and introduce a Turán type mean inequality for the Kummer confluent *k*-hypergeometric function $\phi(z)$.
